# Clinical Outcomes of Voiding Cystourethrogram and Antibiotic Prophylaxis

**DOI:** 10.7759/cureus.46814

**Published:** 2023-10-10

**Authors:** Bassam AlMatrafi, Abdulhakim Al Otay, Ahmed Alhelaly, Mohammed Alhagbani, Abdulrahman Alquliti

**Affiliations:** 1 Department of Pediatric Surgery, Maternity and Children Hospital, Makkah, SAU; 2 Department of Urology, Prince Sultan Military Medical City, Riyadh, SAU; 3 Department of Urology, King Fahad Hospital, Albaha, SAU

**Keywords:** infection, antibiotic, vcug, uti, prophylaxis

## Abstract

Background

Urinary tract infections (UTIs) are a prevalent and potentially serious bacterial infection observed among children. Presently, the primary use of diagnostic imaging for UTI is to pinpoint young patients who are at a high risk of developing renal scarring. The most significant procedure for pediatric urology fluoroscopic evaluation is voiding cystourethrogram (VCUG). VCUG-acquired UTIs continue to be an important concern and the purpose of this study is to assess the clinical outcomes of antibiotic prophylaxis on VCUG-associated UTIs.

Methods

This retrospective study included all patients who underwent VCUG procedures performed from the year 2014 to 2021. All data were retrieved from the medical registries and databases. Radiological and laboratory investigations related to the VCUG procedures were thoroughly reviewed. Patients were considered to have post-procedure UTI if urological symptoms including fever and dysuria along with positive urine culture were exhibited within four weeks after the VCUG study. Patients with incomplete medical records were excluded from the analysis.

Results

This study consisted of 147 participants. Continuous antibiotic prophylaxis (CAP) was observed in 57 (38.8%) participants of them 35 (23.8%) participants suffered from UTI while urine culture and sensitivity testing were performed among 142 (96.6%) participants before VCUG which came negative and only five (3.4%) had a positive result. Overall, the results of the Chi-square test of association revealed a significant association between sex and vesicoureteral reflux (VUR), indicating that the prevalence of VUR differs between males and females. However, no significant associations were observed between VUR and UTI, urine culture and sensitivity results, hydronephrosis, type of catheter, or choice of antibiotic.

Conclusion

In conclusion, this study contributes valuable insights into the clinical outcomes of antibiotic prophylaxis on VCUG-associated UTIs. Despite the prophylaxis rate of 38.8%, UTIs were still observed in a significant proportion of children undergoing VCUG. This calls for further research to identify additional risk factors, optimize prophylaxis strategies, and enhance the overall safety and efficacy of VCUG procedures in children.

## Introduction

Urinary tract infections (UTIs) are a prevalent and potentially serious bacterial infection observed among children and it is reported that by the age of seven, 2% of boys and 8% of girls suffer from UTIs. The frequency of a UTI in febrile children with no other obvious reason is about 7% while 25%-40% of children who are diagnosed with febrile UTIs also have vesicoureteral reflux (VUR). The conventional opinion in the past was that children with any degree of VUR were at a significantly higher risk of developing recurrent pyelonephritis and secondary renal injury, which could lead to scarring, hypertension, and end-stage renal disease. This prompted diagnosis and treatment for all [1,2].

Presently, the primary use of diagnostic imaging UTI is to pinpoint young patients who are at a high risk of developing renal scarring. The possibility of renal scarring has been linked to VUR. As a result, there is debate over the best method for radiologically evaluating children who have UTIs. A diagnostic procedure referred to as voiding cystourethrogram (VCUG) is utilized to precisely detect the presence of VUR as well as its severity. According to retrospective analyses of children with primary VUR, patients with high-grade VUR have a 4-6 times higher risk of scarring than those with low-grade VUR and an 8-10 times higher risk than those without VUR [3]. The most significant procedure for pediatric urology fluoroscopic evaluation is VCUG. It is a dynamic procedure that displays how the lower urinary system functions and integrates. The evaluation of VUR presence or absence is the primary reason for performing VCUG. However, it is also indicated in cases of congenital abnormalities of the urinary tract, such as anorectal malformations, myelodysplastic syndromes, prune-belly syndrome, febrile UTIs, particularly if recurrent, hydronephrosis (HDN) or hydroureter, hematuria, abnormal voiding patterns, such as dysuria, dysfunctional voiding, such as neurogenic bladder, trauma, incontinence, neonatal ascites, and postoperative evaluation of the urinary tract [4].

The majority of VCUG-related complications are not life-threatening, and iatrogenic implications are quite uncommon. Still, it is crucial to be aware of the rare adverse effects that may occur which include bladder rupture, UTIs, and bacteremia [5]. The reported incidence of VCUG-acquired UTI varies and may account for 30% of instances. Since there is a theoretical possibility of bringing periurethral bacteria into the bladder and, in the case of VUR, even into the kidney, performing VCUG with antibiotics prophylaxis seems to be a logical strategy. However, this practice has been questioned as a result of the paucity of strong clinical evidence [6]. The purpose of this study is to assess the clinical outcomes of antibiotic prophylaxis on VCUG-associated UTIs.

## Materials and methods

This retrospective single-center study included all VCUG procedures performed from the year 2014 to 2021. After obtaining institutional ethical approval all data were retrieved from the medical registries and databases. Radiological and laboratory investigations related to the VCUG procedures were thoroughly reviewed by the principal investigator and research team. All pediatric patients (age less than 16 years old) who received continuous antibiotic prophylaxis (CAP) were included while patients with other congenital abnormalities and incomplete medical records were excluded from the analysis. VCUG-associated UTI was considered if urological symptoms including fever and dysuria along with positive urine culture occurred within four weeks after the VCUG study.

Statistical analysis

All the analyses and calculations were performed using Statistical Package for Social Science (SPSS, version 26, IBM Corp., Chicago, IL, USA). Descriptive analysis was performed for demographic variables, Clinical outcomes of VCUG, and CAP using frequency and percentages. A chi-square test was performed to see the significant association for the categorical variable. Binary logistic regression was performed to identify the relationship between antibiotic prophylaxis with clinical outcomes while controlling for potential covariates and associated risk factors. The odds ratio (OR) and confidence interval (95% CI) were reported. A P-value less than 0.05 was considered statistically significant.

Ethical statement

This study was conducted in strict adherence to ethical guidelines and was approved by the Institutional Review Board (IRB) of the Prince Sultan Military Medical City. The approval number for this study is E-2142. All protocols, interventions, and data collection methods were executed as approved by the IRB.

## Results

This study comprised a total of 147 participants of which 88 males and 59 were females. All participants in the study population were Arabs. The age distribution of participants included 22 (14.96%) from the age group five days to one month, 49 (33.33%) from age above one month to 11 months, 42 (28.57%) from one year to five years, 26 (17.68%) from six to 10 years, while eight (5.44%) were from 11 to 15 years. The age distribution is divided across various age ranges, with the largest group falling between one month and 11 months of age (Table 1).

**Table 1:** Frequency distribution of demographic characteristics n: numbers, %: percentage

Variables	n (%)
Sex	
Male	88 (59.9)
Female	59 (40.1)
Age	
5 days – 1 month	22 (14.96)
>1 month -11months	49 (33.33)
1 year-5 years	42 (28.57)
6-10 years	26 (17.68)
11-15 years	8 (5.44)

Almost 23.8% of participants suffered from UTI while urine culture and sensitivity testing were positive for 3.6%. Three cases were positive for E. coli and among them, 1 (0.7%) had received prior treatment for it and subsequently tested positive while the other participant was initially negative but got positive later on repeat sample. VUR was reported in 93 (63.3%) of the sample while HDN was observed in 49 (33.3%) cases. Amoxicillin at a dosage of 5mg/kg/day was received by 14 (9.5%) participants while Bactrim at a dosage of 2mg/kg/day was administered to 43 (29.3%) participants. These clinical characteristics in detail are illustrated in Table 2.

**Table 2:** Frequency distribution of clinical outcomes of voiding cystourethrogram (VCUG) and continuous antibiotic prophylaxis n: numbers; %: percentage; UTI: urinary tract infection; Urine cs:  Urine culture and sensitivity; VUR: vesico-ureteric reflux; HDN: hydronephrosis; fr: French size

Variables	n (%)
UTI	
Yes	35 (23.8)
No	112 (76.2)
Urine CS	
Negative	142 (96.6)
Positive	5 (3.4)
VUR	
Yes	93 (63.3)
No	54 (36.7)
HDN	
Yes	49 (33.3)
No	97 (66)
Type of catheter	
The urethral catheter 6 fr	29 (19.7)
The urethral catheter 8 fr	118 (80.3)
Continuous Antibiotic Prophylaxis	
Yes	57 (38.8)
No	90 (61.2)
Antibiotic	
Amoxicillin 5mg /kg/day	14 (9.5)
Bactrim 2mg/kg /day	43 (29.3)

The sub-group analysis revealed that among individuals without VUR, 62 (66.7%) were male, and 31 (33.33%) were female. While, among individuals with VUR, 26 (48.1%) were male, and 28 (51.9%) were female (p-value = 0.027). Among individuals without VUR, 20 (21.5%) cases had UTI while among individuals with VUR, 15 (27.8%) suffered from UTI (p-value = 0.389). HDN was detected in 28 (30.1%) patients without VUR and in 21 (38.9%) patients with VUR (p-value = 0.212). Among the participants without CAP (n=90), 25.6% had no VUR, and 74.4% had VUR. In contrast, among those with CAP (n=57), 54.4% had no VUR, and 45.6% had VUR. In the VUR group amoxicillin was administered to seven (22.6%) participants while Bactrim was administered to 24 (77.4%) whereas, among individuals without VUR, 19 (73.1%) participants received Bactrim and seven (26.9%) received amoxicillin. Overall, the results of the Chi-square test of association reveal a significant association between sex and VUR, indicating that the prevalence of VUR differs between males and females. However, no significant associations were observed between VUR and post-VCUG UTI. Results of the Chi-square test of the association between VUR and various clinical factors in detail are depicted in Table 3.

**Table 3:** Chi-square test of association with clinical outcomes of voiding cystourethrogram (VCUG) of VUR and other factors. n: numbers; %: percentage; *: p-value < 0.05 shows statistical significance; CAP: continuous antibiotic prophylaxis; UTI: urinary tract infection; Urine cs:  Urine culture and sensitivity; VUR: vesico-ureteric reflux; HDN: hydronephrosis; fr: French size

Variables	(VUR)		P-value
	No	Yes	
	n (%)	n (%)	
Sex			0.027*
Male	62 (66.7)	26 (48.1)	
Female	31 (33.3)	28 (51.9)	
UTI			0.389
Yes	20 (21.5)	15 (27.8)	
No	73 (78.5)	39 (72.2)	
HDN			0.212
Yes	28 (30.1)	21 (38.9)	
No	65 (69.9)	32 (59.3)	

There is a significant association between UTI and CAP (p = 0.031). Among those without UTI, 82.2% did not receive CAP while among those with UTI, 17.8% did not receive CAP. Overall, the results indicate that gender, UTI, and HDN are significantly associated with the clinical outcomes of VCUG with a p-value<0.05. These results are explained in detail in Table 4.

**Table 4:** Chi-square test of association with clinical outcomes of voiding cystourethrogram (VCUG) and antibiotic prophylaxis n: number; %: percentage; *: p-value < 0.05 shows statistical significance; CAP: continuous antibiotic prophylaxis; UTI: urinary tract infection; Urine cs:  Urine culture and sensitivity; VUR: vesico-ureteric reflux; HDN: hydronephrosis; fr: French size

Variables	Continuous antibiotic prophylaxis (CAP)		P-value
	No	Yes	
	N (%)	N (%)	
Sex			0.005*
Female	28 (31.1)	31(54.40)	
Male	62 (68.9)	26 (45.6)	
UTI			0.031*
No	74(82.2)	38(66.7)	
Yes	16 (17.8)	19(33.3)	

The results revealed that HDN emerged as a significant risk factor for post-procedural UTIs, with patients exhibiting approximately 3.4 times higher odds (95% CI; 1.323-8.884; p = 0.011) of developing UTIs compared to those without HDN. On the other hand, no statistically significant association was observed between VUR and the likelihood of post-procedural UTIs, indicating that VUR may not be a significant risk factor for this outcome (p = 0.676). Additionally, sex was found to have no significant impact on the occurrence of UTIs following procedures (p = 0.397). However, when examining different age categories, we found that patients between six and 10 years of age and those above 10 years of age demonstrated a significantly higher likelihood of experiencing post-procedural UTIs compared to the reference age category (five days to one month). For these age groups, the odds of developing UTIs were approximately 8.9 times (95% CI: 1.016-78.432; p = 0.048) and 9.9 times (95% CI; 1.099-90.041; p = 0.041) higher, respectively. Nevertheless, it is important to note that the ORs for some of the age categories were not statistically significant, suggesting that age may not be a universal risk factor for post-procedural UTIs (Table 5).

**Table 5:** Logistic regression for post-procedural UTI with associated risk factors *: p-value < 0.05 shows statistical significance; OR: Odds Ratio; CI: Confidence interval; VUR: vesico-ureteric reflux; HDN: hydronephrosis

	OR	95% CI	P-value
VUR (reference No)	1.207	0.500-2.919	0.676
HDN	3.428	1.323-8.884	0.011
SEX (reference No)	0.682	0.281-1.656	0.397
AGE (reference: 5 days – 1 month)		-	
1 year-5 years	6.405	0.755-54.331	0.089
6-10 years	8.926	1.016-78.432	0.048*
>10 years	9.948	1.099-90.041	0.041*

## Discussion

The study included a retrospective analysis of all VCUG procedures performed over a period of seven years, encompassing a total of 147 participants. Notably, almost 38.8% study participants were on CAP, however, despite the significant prophylaxis rate, the study observed a 23.8% prevalence of UTIs following VCUG, indicating that CAP may not have a role in the prevention of UTI complications associated with the procedure.

The percentage of post-procedural UTI observed in our study is quite high as compared to studies in literature as results of a large-scale retrospective study demonstrated that the incidence of post-procedure UTI following a cystogram is remarkably low almost 1% and post-procedure UTI was only reported in children with prior urologic diagnosis. Therefore, the authors further recommended that peri-procedural antibiotic prophylaxis for children receiving a cystogram should not be given for the primary aim of post-procedure UTI prevention [7]. Similarly, Kalra et al. reported that the risk of a positive culture UTI post-VCUG when bladder catheterization was performed under strict aseptic conditions was quite low among, the children who were already receiving prophylactic antibiotics, negating the need for additional antibiotics before the procedure [8]. Contrarily, findings of a randomized controlled trial demonstrated that the incidence of symptomatic UTI acquired by VCUG, and the incidence of a positive urine culture were considerably greater in patients who underwent VCUG without the use of antibiotics [6].

The percentages in the literature for VCUG-associated UTI vary and are dependent on a multitude of factors including the use of prophylactic antibiotics and the population selection criteria used by different research studies. According to certain research studies, there is a modest risk of developing a UTI following the VCUG, but a significant risk is demonstrated by other research studies findings. As most children who undergo VCUG have serious disorders affecting their urinary system, and developing a UTI following the procedure would worsen their condition [9].

Raed et al. narrated that numerous studies have demonstrated that the lower urinary tract is the cause of UTIs; as a result, catheterization carried out during VCUG can transmit bacteria retrogradely to the upper urinary tract. Authors further reported that 5.76% of patients experienced post-procedure UTI, and all the children had both clinical symptoms and a positive urine culture. This is in accordance with the findings of numerous other research studies that catheterization used during VCUG can retrogradely transfer bacteria to the upper urinary tract. Furthermore, 9/11 children had abnormal cystograms initially, supporting the idea that post-procedure UTI is highly correlated with the presence of a pre-existing urologic condition like VUR or hydronephrosis [10]. Similarly, Rachmiel et al. demonstrated in their findings that the presence of VUR and the severity of VUR were risk factors for the development of UTI after VCUG. Children undergoing prophylactic antibiotic medication have a very low incidence of VCUG-induced UTIs. With only one incidence of E. coli UTI and four cases of Pseudomonas aeruginosa UTI, there is a comparatively high rate of Pseudomonas UTI, particularly in children who have moderate to severe reflux [11].

Our study observed that 33.3% of cases had HDN, while 63.3% experienced VUR. This high prevalence of VUR and HDN may be a contributing factor to the increased occurrence of VCUG-associated UTIs. It suggests that the presence of pre-existing urologic conditions, such as VUR or HDN, is closely linked to the development of UTIs following the procedure. Furthermore, the results of our regression analysis showed that HDN emerged as a significant risk factor for post-procedural UTIs, with patients exhibiting approximately 3.4 times higher odds (95% CI; 1.323-8.884; p =0.011) of developing UTIs compared to those without HDN. This finding aligns with the research conducted by Johnson et al., who also reported that only one child with a normal cystogram developed a UTI after the procedure [7]. On the other hand, no statistically significant association was observed between VUR and the likelihood of post-procedural UTIs.

In clinical practice, VUR and HDN are observed to be responsible for UTIs as results of a comparative study showed that a younger mean age at the first UTI, bilateral reflux, grade 4-5 VUR, and HDN on the initial ultrasound scan all significantly elevated the probability of recurrent UTI. High-grade and bilateral VUR were independently linked with an elevated likelihood of recurrent UTI in multivariate analysis. The delayed contrast passing on the VCUG revealed that 80% and 0% of the young children with and without recurrent UTI had high-grade VUR, respectively [12]. Additionally, Chang et al. stated that recurrent UTIs are more common in children with VUR [13]. Furthermore, Estrada et al. described that diagnosis of VUR, and use of prophylactic antibiotics dramatically lower the risk of febrile UTI in patients with a history of prenatal HDN who are observed to have postnatally persistent grade II HDN. As a result, patients with a history of prenatal HDN or postnatally persistent HDN should be examined early in life with VCUG and placed on prophylactic antibiotics until the screening results have been determined [14]. However, the use of CAP in the prevention of VCUG-associated UTI still remains controversial and debatable necessitating the need of further research.

Sinha et al commented that in pediatric radiology, VCUG is a routine fluoroscopic examination. Even while recent recommendations aimed to limit the use of VCUG in children with UTI, numerous studies have cast doubt on these recommendations and demonstrated higher chances of missing important underlying lesions. There is strong agreement regarding the usage of antibiotics, even though VCUG is frequently used in pediatric practice. Even the recently acknowledged practice parameter for VCUG by the American College of Radiology Society for Paediatric Radiology is silent on its use. Some international guideline does advocate the use of antibiotics for VCUG, but the lack of strong evidence has led to questions about the benefits of this strategy [6].

Individuals with UTI had a lower likelihood of receiving antibiotic prophylaxis and this association was statistically significant. Autore et al. described that the likelihood of UTI recurrence does not appear to be affected by long-term antibiotic prophylaxis, and it is not usually recommended. When performing VCUG which is indicated in children who have a history of recurrent UTIs with three episodes/year or who have VUR grade IV-V, antibiotic prophylaxis may be considered up until the procedure is completed. Third generation cephalosporins or oral amoxicillin/clavulanic acid are also viable alternatives for long-term prophylaxis. The best compounds, dosages, and duration for long-term antibiotic prophylaxis are not yet clear from the available data. In general, doses between one-third and 50% of those given during the acute infection are thought to be acceptable for long-term prophylaxis [15]. Our study is one of the few available to our knowledge that discusses the use of antibiotic prophylaxis, particularly in relation to VCUG-associated UTIs among children. This represents a significant strength of our study. However, it is essential to acknowledge that the literature on post-procedure UTIs is scarce and limited, which makes it challenging to extensively compare our findings with other studies.

While our study provides valuable insights into the clinical outcomes of VCUG and associated UTIs, we recognize certain limitations that should be taken into account when comparing our results to other studies. The retrospective design of our study might introduce biases and limit our ability to draw causal conclusions. Moreover, the relatively small sample size and homogeneity of our study population in terms of ethnicity may restrict the generalizability of our findings to more diverse populations. To enhance the robustness and applicability of the results, future studies could consider employing a prospective design with larger and more diverse samples. A well-designed randomized controlled trial with proper randomization and blinding could provide stronger evidence on the effectiveness of antibiotic prophylaxis in preventing UTIs after VCUG. Furthermore, conducting subgroup analyses based on patient demographics, comorbidities, and different antibiotic regimens could offer valuable insights into potential variations in prophylactic efficacy. Understanding which patient factors influence the success of antibiotic prophylaxis can help tailor treatment strategies for specific populations.

## Conclusions

In conclusion, this study contributes valuable insights into the clinical outcomes of antibiotic prophylaxis on VCUG-associated UTIs which can be utilized in future research studies and can aid in the development of evidence-based guidelines and recommendations for clinical practice. Despite the increased prophylaxis rate, UTIs were still observed in a significant proportion of children undergoing VCUG. Nevertheless, CAP has a significant role in reducing VCUG-associated UTIs. This calls for further research to identify additional risk factors, optimize prophylaxis strategies, and enhance the overall safety and efficacy of VCUG procedures in children.
